# Evaluation of canine intervertebral disc degeneration in colour-coded computed tomography

**DOI:** 10.1186/s13620-015-0054-1

**Published:** 2015-11-11

**Authors:** Lisa K. Harder, Vladimir Galindo-Zamora, Martin Beyerbach, Ingo Nolte, Patrick Wefstaedt

**Affiliations:** Small Animal Hospital, University of Veterinary Medicine Hannover, Foundation, Bünteweg 9, D-30559 Hannover, Germany; Small Animal Clinic, Faculty of Veterinary Medicine, National University of Colombia, Carrera 30 45-03 (Ciudad Universitaria), Bogotá, Colombia; Institut for Biometry, Epidemiology and Information Processing, University of Veterinary Medicine Hannover, Foundation, Bünteweg 2, D-30559 Hannover, Germany

**Keywords:** Computed tomography, Dog, Canine, Colour, Intervertebral disc degeneration, Classification, Comparison, Magnetic resonance imaging

## Abstract

**Background:**

Canine intervertebral disc degeneration can lead to intervertebral disc disease. Mild degenerative changes in the structure of the canine intervertebral disc can be identified in magnetic resonance images, whereas these changes are not visible in computed tomographic images. Therefore, one aim of this study was to detect whether colour-coded computed tomography enhances the visibility of mild degenerative changes in the canine disc structure compared to non-contrast computed tomography. Furthermore, the study aimed to detect if intervertebral disc degeneration could be classified with a higher reliability in colour-coded images than in non-contrast images.

**Results:**

Computed tomographic image studies of 144 canine intervertebral discs were coloured using three different lookup tables. Canine intervertebral disc degeneration was evaluated by three observers using a 5-grade classification system and compared to the evaluation of non-contrast CT and MRI images. A moderate to almost perfect intraobserver and a moderate to substantial interobserver agreement were found depending on the used colour code. On comparing non-contrast and colour-coded CT significant differences were found by one observer only. Significant differences in evaluation were found in grading intervertebral disc degeneration in MRI and colour-coded CT.

**Conclusions:**

Intervertebral disc degeneration could not be classified with a higher reliability on colour-coded images compared to non-contrast images. Furthermore, colour-coded CT did not enhance the visibility of mild degenerative changes in disc structure compared to non-contrast CT.

However, the better intraobserver agreement and the subjective impression of the observers highlighted that the usage of colour encoded CT data sets with a wide range of tonal values of few primary and secondary colours may facilitate evaluation.

## Background

Canine intervertebral disc (IVD) disease is a frequent result of IVD degeneration [1, 2]. Diagnostic imaging is a useful tool to detect degenerative changes in IVDs [3]. Magnetic resonance imaging is said to be the gold standard in the classification of IVD degeneration in human and veterinary medicine, detecting morphological changes as well as biochemical changes in disc composition [4, 5]. However, equipment acquisition costs and special training requirements of the staff restrict the usage of magnetic resonance imaging in veterinary medicine. In contrast, computed tomography (CT) is more readily available and easier to perform. Several studies have confirmed the benefit of using CT examinations to show anatomical structures of the canine spine [6, 7]. Furthermore, CT imaging of the IVD and displaced disc material have been reported [8–10]. Recently, the authors of this study suggested a grading system for evaluating IVD degeneration in CT [11]. In comparison to T2 weighted magnetic resonance imaging, initial degenerative changes in IVD morphology were not seen in non-contrast CT images [11]. CT images consist of Hounsfield Units, which are depending on the attenuation of the exposed structure and tissue [12]. These Hounsfield Units determine a tonal value from white to black in the native CT image. As a result, small differences in grey values are difficult to detect for the human eye which can distinguish only 20 grey values simultaneously [13]. However, more than 1,600,000 tonal values of several colours can be separated from each other [13, 14]. In this context we hypothesise that the visibility of IVD structures in non-contrast transverse CT studies can be enhanced for the human eye when images are colour-coded instead of being displayed as a grey scale image.

Therefore, one aim of the present study was to test whether the colour-encoding enhances intra- and interobserver agreement in the evaluation of canine IVD degeneration. Furthermore, the study aimed to detect whether colour-coded CT ameliorates the visibility of early degenerative changes in the canine IVD structure compared to non-contrast CT.

## Methods

MRI and CT records of client-owned dogs which were presented at the Small Animal Clinic, University of Veterinary Medicine Hannover, Foundation between April 2011 and March 2012 were reviewed in this retrospective study. Cases were included if MRI and CT records of the same intervertebral disc spaceswere available, data sets were obtained on the same day and the dogs were suspicious of having spinal cord compression. Data sets of 43 client-owned dogs met the inclusion criteria^1^. The patients had a mean age of 6.2 years (from 5 months up to 14 years) and a mean weight of 15.9 kg (from 3.8 to 60 kg). CT records were obtained with a third generation 64-detector row computed tomography system^2^. The cervical and the thoracic vertebral column were scanned with 1.5 mm slice thickness, 120 kV voltage and a current of 200 mAs (cervical spine) per slice and 250 mAs/slice (thoracic spine), respectively. The lumbar vertebral column was scanned with 2 mm slice thickness, 140 kV voltage and a current of 200 mAs/slice. Reviewed MRI records were transverse T2 weighted Fast Field Echo images (Time to echo 120 ms, repetition time 4.5–12.2 s, slice thickness 1.8–5 mm) obtained in a 3.0 T Highfield MRI^3^. Analysis of IVD degeneration in non-contrast CT (using a self-developed classification system) and MRI (using the Pfirrmann scoring system) had already been carried out as part of another study using identical non-contrast MRI and CT data sets from the same dogs [11]. The results of these evaluations were used for comparison with the colour-coded analysis carried out in the study described here.

### Digital image processing of CT data sets

**Fig. 1:**
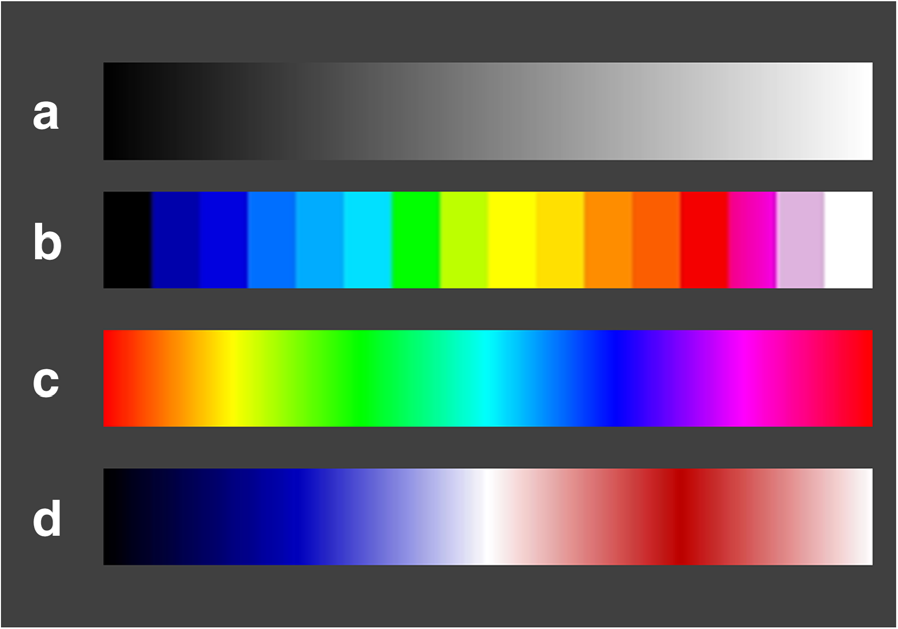
Colour-lookup-tables for grey value replacement. **a** Grey Values; **b** Colour 16; **c** Spectrum; **d** Union Jack

Acquired non-contrast CT DICOM data sets were exported to ImageJ^4^. All image studies were edited using three different lookup-tables of the program (Colour16, Spectrum, Union Jack) and saved as 8 bit RGB-TIF. These lookup-tables create no RGB-Image but replace grey values with deposited colours. The three lookup-tables chosen contained different numbers of tonal colour values as seen in Fig. 1. While Colour16 includes 16 colours, Spectrum includes 256 tonal values of all basic colours and Union Jack includes 191 tonal values of the colours black, white, red and blue. Brightness and contrast of Spectrum were standardised in all images setting a range of 33 to 288 with the ImageJ tool “brightness”. All data sets were doubled, blinded and randomised for evaluation.

### Evaluation

**Fig. 2:**
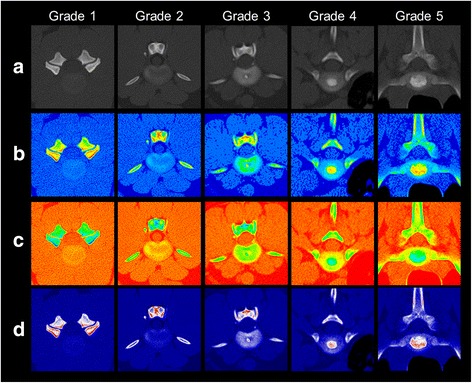
Classification of intervertebral disc degeneration in non-contrast and colour-coded computed tomographic images. Classification of disc degeneration in non-contrast and colour-coded computed tomography. The columns show intervertebral discs as example for the grading of disc degeneration in computed tomographic imaging. Each column shows the same intervertebral disc as non-contrast image and as a coloured image of each method. The rows show examples of all 5 grades of degenerative changes in non-contrast (**a**) and Colour_16 (**b**), Spectrum (**c**) and Union Jack (**d**) coloured images

Three observers with different degrees of experience in evaluating computed tomographic images (observer 1, 2 years of experience (LH); observer 2, 5 years of experience (VGZ); observer 3, 24 years of experience (IN)) evaluated all images on standard computer screens using ImageJ. A self-developed scoring system was used to evaluate IVD degeneration (Table 1) [11]. The observers were familiarised with the scoring system, being given several examples of the various grades of IVD degeneration in colour-coded images (Fig. 2). However, the observers had no training session together and did not discuss the grading with each other before evaluation. Additionally, ten data sets of canine IVDs colour-coded with the three different lookup-tables were available for test evaluation purposes. These training data sets were not part of the study. After familiarising themselves with the scoring system, each observer evaluated all blinded and randomised images twice. One dataset included several IVDs which were graded separately.

**Table 1:** Classification of intervertebral disc degeneration

Grade	Structure	Distinction between NP and AF	Attenuation	Height of IVD
1	Homogeneous	Lost	Isodense to M. long. dorsi	Normal
2	Homogeneous	Slightly-clearly visible	NP isodense to M. long. dorsi; AF hyperattenuating to M. long. dorsi	Normal
3	Nonhomogeneous	Clearly visible	One dot-like homogeneneous, hyperattenuating Mineralisation often within the NP; AF iso-/hyperattenuating to M. long. dorsi	Normal to slightly decreased
4	Nonhomogeneous	Clear or hidden by mineralised material	Multiple small or one bigger hyperattenuating Mineralisation, which cover up to 1/3 of the IVD AF iso-/hyperattenuating to M. long. dorsi	Normal to moderatly decreased
5	Nonhomogeneous	Hidden by mineralised material	Hyperattenuating, inhomogeneous Mineralisation which cover at least 1/3 of the IVD; diffus hyperattenuating to M. long. dorsi; AF iso-/hyperattenuating to M. long. dorsi	Collapsed disc space

Self-developed grading system to classify canine intervertebral disc degeneration

*Abbreviations*: *AF* Anulus fibrosus; *IVD* Intervertebral disc; *M. long. dorsi* Musculus longissimus dorsi; *NP* Nucleus pulposus

### Statistical analysis

Weighted Kappa Analysis was performed in SAS^5^ to obtain intraobserver and interobserver agreement in all three colour-coded methods and to compare the evaluation of the non-contrast and the three colour-coded data sets. Furthermore, magnetic resonance imaging and colour-coded images were compared using the same statistical tests. Agreement was interpreted according to Landis and Koch as being slight (ĸ 0–0.20), fair (ĸ 0.21–0.4), moderate (ĸ 0.41–0.6), substantial (ĸ 0.61–0.8) and excellent (ĸ 0.81–1) [15]. Bowker’s test was used to detect differences in evaluation.

## Results

### Intra- and interobserver agreement of IVD degeneration in colour-coded CT images

The evaluation of IVD degeneration yielded a moderate to substantial intraobserver agreement in Colour16 images, substantial agreement in Spectrum images and substantial to almost perfect agreement in Union Jack images (Table 2).

**Table 2:** Intra- and Interobserver agreement in non-contrast and colour-coded CT-images

Comparison κ	Non-contrast	Colour 16	Spectrum	Union Jack
Intraobserver				
1	0.81^a^	0.74	0.78	0.81
2	0.74^a^	0.59	0.62	0.67
3	0.62^a^	0.75	0.72	0.76
Interobserver				
1 and 2	0.68^a^	0.55	0.43	0.52
1 and 3	0,69^a^	0.71	0.72	0.68
2 and 3	0.60^a^	0.63	0.51	0.62

Intraobserver agreement between two grading sessions and interobserver agreement between three observers. ^a^Source: Tierärztliche Praxis

Interobserver agreement was moderate to substantial in the three colour-coded methods. However, the results showed differences in evaluation among the observers (Table 2). A moderate agreement was found between observers 1 and 2 in all methods, substantial agreement between observers 1 and 3 and a moderate to substantial agreement between observers 2 and 3. Compared to the intra- and interobserver agreement in non-contrast CT a slightly lower agreement was achieved in the evaluation of colour-coded images.

### Comparison of colour-coded methods

**Fig. 3:**
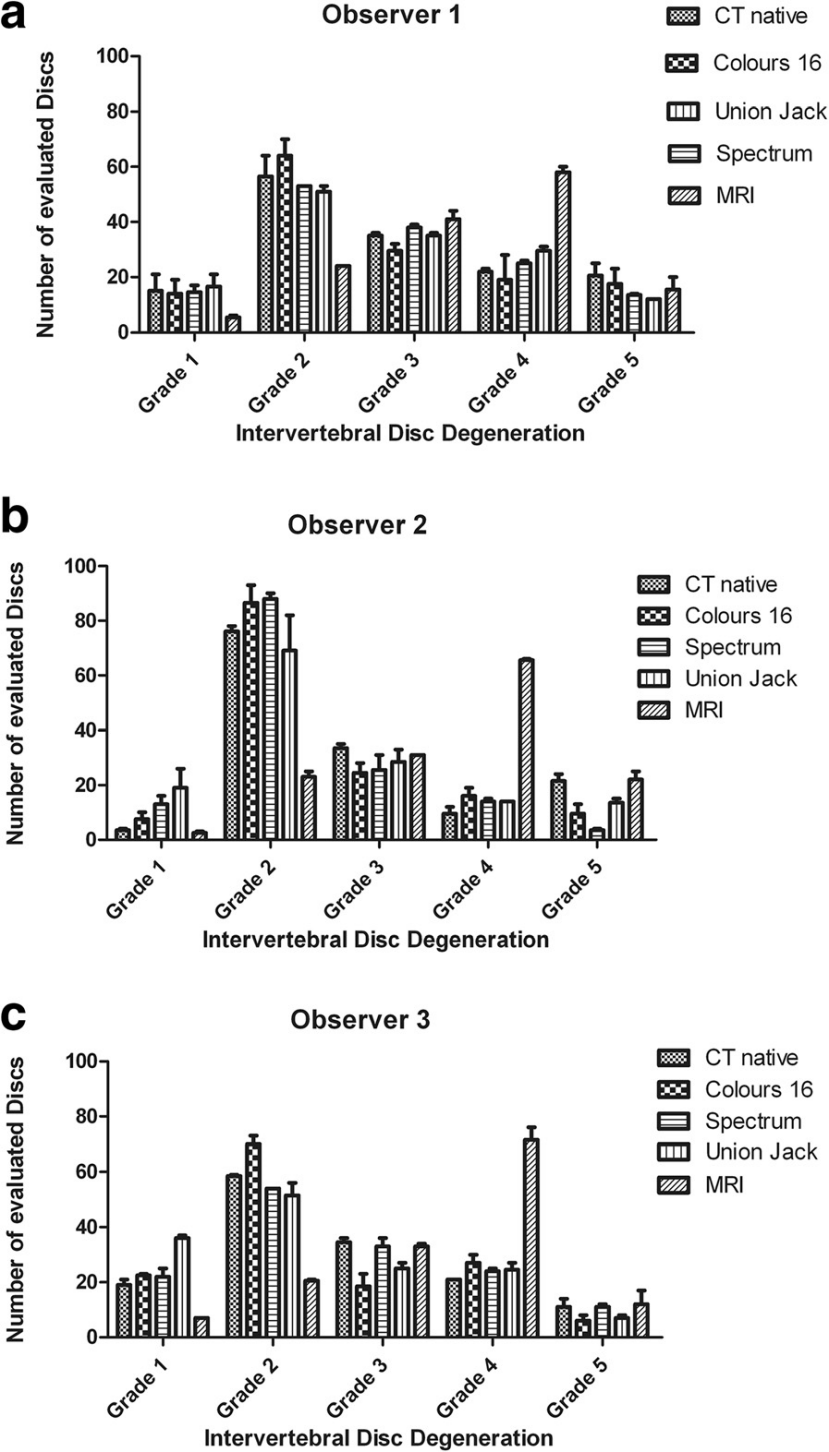
Evaluation of intervertebral disc degeneration in non-contrast and colour-coded computed tomographic images. Mean value and standard deviation of the 2 evaluation sessions of observer 1 (**a**), observer 2 (**b**) and observer 3 (**c**). Significant differences in evaluation were found between evaluation of non-contrast CT images and spectrum and union jack coloured images in observer 2

Comparison of Colour16- and Spectrum-coloured images yielded substantial agreement, while the comparison of Union Jack images with Colour16 images and Spectrum images showed moderate to substantial agreement (Table 3). Limits of confidence showed a range of 0.12 to 0.23, being very small (0.12–0.14) from observer 1 and wider (0.13–0.23) from observers 2 and 3. One-sided p-values to Kappa showed high correlation in evaluation of colour-coded images (*p* < 0.0001). Bowker’s test showed no significant differences in evaluation of IVD degeneration. The similar results of evaluation of the different colour-coded images can be seen in Fig. 3. The subjective impression of all observers was that evaluation was easier to perform in the Union Jack than in Colour16-coded images, followed by Spectrum images.

**Table 3:** Comparison of coloured data sets

Comparison	Observer	ĸ	Limits of confidence	*p*-value of Bowker’s test
B16:Spectrum	1	0.71	0.64–0.78	0.54
	2	0.62	0.51–0.73	0.34
	3	0.66	0.59–0.74	0.48
B16:Union Jack	1	0.78	0.72–0.84	0.9
	2	0.60	0.51–0.69	0.89
	3	0.75	0.69–0.82	0.27
Spectrum: Union Jack	1	0.77	0.71–0.84	0.98
	2	0.60	0.50–0.70	0.38
	3	0.72	0.56–0.79	0.12

Bowker’s test shows no significant differences in evaluation of IVD degeneration

### Comparison of non-contrast and colour-coded classification of IVD degeneration

Comparison of non-contrast and Colour16 images and Spectrum images yielded a moderate to substantial agreement, while agreement between non-contrast and Union Jack images showed substantial agreement among all three observers (Table 4). The corresponding limits of confidence varied within a range of 0.14 to 0.20 depending on the observer. The *p*-value of the weighted kappa test showed a significant correlation in evaluation, being smaller than 0.0001. All three observers achieved no significantly different results in the evaluation of non-contrast and Colour 16 images analysed in Bowker’s test. Furthermore, the comparison of non-contrast CT images with Spectrum and Union Jack images from observers 1 and 3 was not significantly different, while significant differences in evaluation were found from observer 2 between non-contrast and Spectrum and non-contrast and Union Jack-coloured images. These differences in the evaluation of observer 2 can be seen in Fig. 3b). More colour-coded images were evaluated as being healthy grade 1 IVDs compared to the evaluation of non-contrast CT images. Slightly more grade 2 IVDs were seen in Colour16 and Spectrum than in non-contrast CT. Slightly less grade 2 IVD degeneration was seen in Union Jack images compared to non-contrast CT. Observer 2 graded more IVDs in non-contrast images as grade 3 than in colour-coded images. Slightly more grade 4 IVDs were found in the colour-coded images than in non-contrast CT data. Fewer grade 5 IVDs were found in colour-coded methods than in non-contrast data.

**Table 4:** Comparison of non-contrast and coloured data sets

Comparison	Observer	ĸ	Limits of confidence	*p*-value of Bowker’s test
Non-contrast : B16	1	0.73	0.72–0.86	0.57
	2	0.57	0.47–0.67	0.21
	3	0.67	0.60–0.74	0.61
Non-contrast : Spectrum	1	0.74	0.67–0.81	0.92
	2	0.51	0.41–0.61	<0.0001***
	3	0.67	0.59–0.74	0.77
Non-contrast : Union Jack	1	0.76	0.69–0.83	0.84
	2	0.66	0.58–0.74	0.023**
	3	0.62	0.55–0.69	0.10

Bowker’s test shows significant differences in evaluation of IVD degeneration between non-contrast and coloured images in observer 2 with p-value: **p < 0.01, ***p < 0.001

The normal IVD can be easily distinguished from the surrounding tissue in Colour 16 images (Fig. 2, Grade 1 b). Grade 2 IVDs show a good contrast between anular and nuclear material (Fig. 2, Grade 2 b). Mineralised IVD material consists of orange and yellow tonal values in Colour16 which differs greatly from the blue tonal values of the normal IVD material (Fig. 2, Grade 3–5 b). The red and orange tonal values of hyperattenuating structures contrast strongly with the surrounding, yellow mineralised IVD tissue (Fig. 2, Grades 4 and 5). Hypoattenuating areas within the mineralisation, as seen in the Colour16 grade 5 disc (Fig. 2) are lime green and have a poor contrast to the yellow mineralisation. If these lime green areas are next to cyan blue IVD material (Fig. 2, Grade 5) the silhouette of the mineralisation can hardly be seen. At first glance, the mineralisations seem to have a smaller extension in Colour16 than in non-contrast CT.

In Spectrum images the IVD shows several yellow, orange and red tonal values. IVD material can be better differentiated from the surrounding tissue in Spectrum images than in non-contrast CT. Hyperattenuating annular regions show more yellow tonal values and can be identified easily. Setting disc mineralisations apart from IVD material is difficult due to a high brightness of the colours. Especially in the periphery of mineralisations lime green tonal values make the contour to the yellow IVD material poorly defined. Therefore, the mineralisations are optically larger in size in Spectrum images than in non-contrast CT.

In Union Jack images, dark blue IVD material is hard to distinguish from the surrounding tissue. Slight changes in attenuation in the periphery of the IVD are easy to see as white, cloudy areas of white and light blue pixels within the dark blue disc material. Mineralisations are seen as bright white regions in the dark blue disc with a clear silhouette. Hyperattenuating parts of mineralised IVD material are red and contrast strongly with the white colours.

All observers reported that the changes could be seen more easily in Union Jack-coloured images than in Colour 16 and Spectrum images.

### Comparison of colour-coded CT and MRI-images

Comparison of MRI and colour-coded CT images yielded a slight to fair intraobserver agreement with the following weighted kappa coefficients and associated limits of confidence: Colour 16 and MRI 0.34 (0.25–0.43; LH), 0.15 (0.007–0.23; VGZ) and 0.19 (0.12–0.26; IN), respectively; Spectrum and MRI 0.31 (0.23–0.39; LH), 0.13 (0.07–0.19; VGZ), 0.21 (0.13–0.28; IN), respectively; Union Jack and MRI 0.31 (0.23–0.4; LH), 0.20 (0.11–0.28; VGZ), 0.22(0.15–0.3; IN), respectively. Statistical analysis using Bowker’s test showed significant differences in evaluation of intervertebral disc degeneration in all colour-coded images compared to evaluation of Pfirrmann Grade in MRI (*p*-value <0.0001).

## Discussion

The aim of the present study was to test whether colour-coded CT images simplify the evaluation of data sets and enhance the visibility of degenerative changes in the structure of the canine IVD. Therefore, the reliability of evaluation of IVD degeneration in colour-coded data was tested and the evaluation of IVD degeneration in non-contrast and colour-coded CT data was compared.

Evaluation of canine IVD degeneration in colour-coded CT images using the proposed grading system is possible due to a predominantly good intra- and interobserver reproducibility [16].

There were no statistically significant differences in the evaluation of IVD degeneration in different colour-coded CT images although the observers preferred the Union Jack coloured images. Furthermore, a higher reliability could be found in the evaluation of Union Jack coloured images compared to the other colour-coded methods. On comparing the lookup-table Colour16 with the other two colour-coded methods, fewer tonal values are used in that method. Thus, a small number of tonal values did not facilitate evaluation. On comparing Spectrum with Union Jack more tonal values of several primary and secondary colours are used in Spectrum while the Union Jack is limited to 191 tonal values of the colours red, blue, white and black. Consequently, a wider range of tonal values may facilitate the evaluation of CT images, while the usage of several primary and secondary colours seems not to provide any additional benefits. In Union Jack coloured images the human eye perceives the different colours with the retinal cones as well as the rods. The grey values of non-contrast CT images are only perceived by the retinal rods [13, 14]. Therefore a smaller number of tonal values can be distinguished simultaneously [13]. Perhaps the Union Jack coloured images were easier to evaluate due to a greater contrast between the tonal values of 4 colours, while in Spectrum coloured images a poor contrast between several bright colours could be seen.

The comparison of the evaluation of IVD degeneration in colour-coded and non-contrast CT images shows that the usage of colours does not enhance the reliability in evaluation compared to the evaluation of non-contrast CT images. This may be a result of the familiarisation of all observers with the non-contrast CT images.

A further aim of this study was to enhance the visibility of IVD degeneration in CT images. Significant differences in evaluation of IVD degeneration in non-contrast and colour-coded CT images were found by one observer. Kovacs et al. defined a cut-off point of normal and abnormal IVD degeneration between Pfirrmann Grades 2 and 3 for the classification of IVD degeneration in MRI images [3, 17, 18]. Our recent comparison of the evaluation of IVD degeneration in non-contrast CT and magnetic resonance images showed that this cut-off point cannot be applied to the CT evaluation of IVD degeneration [11]. In the present study, more Grade 1 and 2 IVDs were found in the evaluation of coloured images than in non-contrast images (Fig. 3). From these results it can be concluded that colour-coded images did not show signs of IVD degeneration at an earlier stage than non-contrast CT. One reason for this result might be that image information may be lost by the image processing with colours. The exchange of tonal values in a black and white image with several colours can be compared to changes in the window level. On the one hand, changing the window level can provide more detailed information by creating a better contrast of the annulus fibrosus and nucleus pulposus; on the other hand, information in other tissue, for example mineralised parts of the intervertebral disc, can be lost. The same problem exists in colour-encoding of images. The visibility of hyperattenuating material in the periphery of the IVD arose in Colour16 and Spectrum, while the size of mineralisationss was most likely to be underestimated in Colour16 and overestimated in Spectrum. Compared to non-contrast CT images, in coloured images some hyperattenuating structures in the anular area of the IVD were easier to see in Colour16 and Spectrum images. Since no pathological and histological examinations of the IVDs could be performed in this study the findings in non-contrastand colour-coded CT-images could not be confirmed. This fact limits the interpretation of the results.

## Conclusion

In conclusion, the colour-encoding does not lead to a better intra- and inter-observer agreement than the evaluation of non-contrast CT images. Furthermore, colour-coded CT did not ameliorate the identification of early degenerative changes in IVD in comparison to non-contrast CT. The three different lookup-tables used in the present study to colour CT images had no statistically significant effect on evaluation when compared with one another. However, the better intraobserver agreement and the subjective impression of the observers highlighted that the usage of colour encoded CT data sets with a wide range of tonal values of few primary and secondary colours may facilitate evaluation.

## Abbreviations

CT: Computed tomography
MRI: Magnetic resonance imaging
IVD: Intervertebral disc

## Footnotes

1 15 Dachshunds, 7 Mixedbreeds, 2 Beagles, 2 Dalmatians, 2 Bernese Mountain Dogs, 2 Jack Russell Terrier, 2 German Shepherd Dogs, 1 Berger de Pyrenées, 1 Bolonka Zwetna, 1 Austrian Black and Tan Hound, 1 English Cocker Spaniel, 1 Hannoverian Scenthound, 1 German Hunting Terrier, 1 Karelian Bear Dog, 1 Labrador Retriever, 1 Pug, 1 Shih Tzu, 1 Sky Terrier

2 Brilliance 64, Philips Medical Systems, Best, the Netherlands

3 Achieva 3.0 T TX, Philips Medical Systems, Best, the Netherlands

4 Image Processing Analysis in Java, National Insitute of Health, Bethesda, USA

5 Statistical Analysis Software, SAS Institute Inc., Cary, North Carolina, USA.
